# Insight into the Prospects for Tumor Therapy Based on Photodynamic Immunotherapy

**DOI:** 10.3390/ph15111359

**Published:** 2022-11-04

**Authors:** Xiaoxia Cheng, Yiqu Wei, Xiaomei Jiang, Chunli Wang, Mengyu Liu, Jiaxin Yan, Lei Zhang, Yaqi Zhou

**Affiliations:** 1 School of Basic Medical Sciences, Henan University, Kaifeng 475004, China; 2 School of Clinical Medicine, Henan University, Kaifeng 475004, China; 3 School of Pharmacy, Henan University, Kaifeng 475004, China; 4 Pathology Department, Jiaozuo Second People’s Hospital, Jiaozuo 454001, China

**Keywords:** photoimmunotherapy, photodynamic therapy, malignant tumor treatment, immunity, REACTIVE oxygen species

## Abstract

Malignancy is one of the common diseases with high mortality worldwide and the most important obstacle to improving the overall life expectancy of the population in the 21st century. Currently, single or combined treatments, including surgery, chemotherapy, and radiotherapy, are still the mainstream regimens for tumor treatment, but they all present significant side effects on normal tissues and organs, such as organ hypofunction, energy metabolism disorders, and various concurrent diseases. Based on this, theranostic measures for the highly selective killing of tumor cells have always been a hot area in cancer-related fields, among which photodynamic therapy (PDT) is expected to be an ideal candidate for practical clinical application due to its precise targeting and excellent safety performance, so-called PDT refers to a therapeutic method mainly composed of photosensitizers (PSs), laser light, and reactive oxygen species (ROS). Photoimmunotherapy (PIT), a combination of PDT and immunotherapy, can induce systemic antitumor immune responses and inhibit continuing growth and distant metastasis of residual tumor cells, demonstrating a promising application prospect. This article reviews the types of immune responses that occur in the host after PDT treatment, including innate and adaptive immunity. To further help PIT-related drugs improve their pharmacokinetic properties and bioavailability, we highlight the potential improvement of photodynamic immunotherapy from three aspects: immunostimulatory agents, tumor-associated antigens (TAAs) as well as different immune cells. Finally, we focus on recent advances in various strategies and shed light on their corresponding mechanisms of immune activation and possible clinical applications such as cancer vaccines. Having discovered the inherent potential of PDT and the mechanisms that PDT triggers host immune responses, a variety of immunotherapeutic strategies have been investigated in parallel with approaches to improve PDT efficiency. However, it remains to be further elucidated under what conditions the immune effect induced by PDT can achieve tumor immunosuppression and to what extent PDT-induced antitumor immunity will lead to complete tumor rejection. Currently, PIT presents several outstanding intractable challenges, such as the aggregation ability of PSs locally in tumors, deep tissue penetration ability of laser light, immune escape, and biological toxicity, and it is hoped that these issues raised will help to point out the direction of preclinical research on PIT and accelerate its transition to clinical practice.

## 1. Introduction

PDT, a minimally invasive and promising new approach to cancer treatment, has been introduced into the therapy of various cancers, such as bladder cancer, lung cancer, head and neck cancers as well as skin cancer [1]. Patient takes a nontoxic photosensitive drug or PS, which absorbs light and stimulates electron transfer when irradiated by the light of specific wavelengths in tumor tissue regions, involving a range of photochemical reactions and highly triggering the conversion of reactive monomorphic oxygen species to ROS. Firstly, the produced ROS directly kills cancer cells through necrosis or apoptosis [2]. Secondly, ROS disrupts the tumor-associated vascular system, which may result in tumor destruction due to oxygen and nutrition deficiency. Thirdly, PDT stimulates and enhances antitumor immunity because it does not only directly kill the cancer cells but also promotes antitumor immune responses through different cellular pathways [3], including triggering acute inflammatory responses, secreting pro-inflammatory cytokines, redistribution, activation, and infiltration of immune effector cells. The mechanisms of these processes are complex, involving almost all the aspects of the immune system, which plays an essential role in preventing metastasis and recurrence of tumors.

Malignant tumor, a serious threat to human health, is a leading cause of death around the world. Despite systemic therapy such as chemotherapy and radiotherapy, there is still poor prognosis and high mortality among cancer patients. Metastasis and recurrence are two thorny issues that threaten the progress in cancer treatment [4]. Tumor is the product of the malignant transformation of normal cells characterized by persistent diffusion and metastasis in vivo. The emergence of new antigen markers is a typical immune feature of tumor cells, which are recognized by the body’s immune defense system and thus stimulate immune responses. Innate immune cells are able to detect tumor antigens, which in turn induce and amplify adaptive immune responses, thereby exerting direct responses [5].

PDT offers several unique advantages over traditional cancer treatment options such as chemotherapy, radiotherapy, and surgery, including non-invasive, low systemic toxicity, precise tumor targeting, significant efficacy, no resistance, and low cost. However, PDT is limited by some clinical disadvantages, such as poor tissue penetration, hydrophobicity, and cutaneous photosensitivity. To reduce adverse reactions associated with PDT and improve the therapeutic effect, more attempts have been made to combine PDT with other traditional cancer therapies such as chemotherapy, radiotherapy, immunotherapy, and enzyme inhibitors [6]. Combined therapy could reduce drug dosage, meaningfully improve the patients’ quality of life, reduce monotherapy-related side effects and increase antitumor effects, thus providing a significant survival benefit.

The ideal cancer therapy should be characterized by destroying the primary tumor and identifying, tracking, and destroying the residual tumor cells [2]. These desirable properties for triggering and enhancing antitumor immune responses are all manifested in PDT. In addition, many immunotherapies have been used in clinical studies of metastatic cancer, including monoclonal antibody (mAb) therapy, cytokine therapy, vaccination checkpoint inhibition, and so on. In particular, the discovery of dendritic cell (DC)-based vaccines and checkpoint inhibitors won the Nobel Prizes in 2011 and 2018. Although cancer immunotherapy has achieved promising results in preclinical and clinical studies of localized tumors, limiting metastasis remains a great challenge [7]. Therefore, the combination of PDT and immunotherapy, namely PIT, an emerging tumor-targeted phototherapy that combines the tumor specificity of monoclonal antibodies with the phototoxicity of light absorbers to induce the immunogenic death of target tumor cells rapidly and selectively [8], which induces a systemic antitumor immune response and control residual tumor cells as well as distant metastasis at the treatment site, thus achieving highly targeted tumor therapy with minimal normal tissue damage, which could significantly improve the efficacy of the treatment and expand its application in clinical practice. In this review, the types of immune responses that occur in the host following PDT, including innate and adaptive immunity, are introduced. Further, we highlight the weaknesses of PIT as well as potential options for improvement.

## 2. PDT Triggers Host Immunity

### 2.1. The Mechanism of Photodynamic Triggering Immunotherapy

As an exploration of new tumor treatment strategies continues, various tumor ablation methods, including ultrasound ablation, cryoablation, and microwave ablation, have been used in the treatment of malignant tumors in the clinic. In addition to the direct killing effect, tumor ablation after tumor cell death, as a potential antigen pool, triggers an immune response [9,10]. PDT is a treatment method in that PS is irradiated by specific light wavelength in tumor tissues to generate ROS, such as peroxides, free radicals, ions, and singlet oxygen [11]. PDT performs direct eliminating tumor by ablation and could induce cytotoxic lymphocytes (CTLs) to exert a sustained antitumor immune effect [12], a phenomenon referred to as PIT, already described in the introduction. Unlike the immunosuppression effect of traditional treatment methods, tumor treatment with PIT significantly enhances the body’s immune response [13]. In PIT, the PS selectively gathers in the target organs, target tissues, and target cells after injection into the body. Additionally, PDT is adjustable within the tumor-microenvironment (TME) immune alterations [12] and plays an important role in the injury or death of tumor cells.

**Figure 1:**
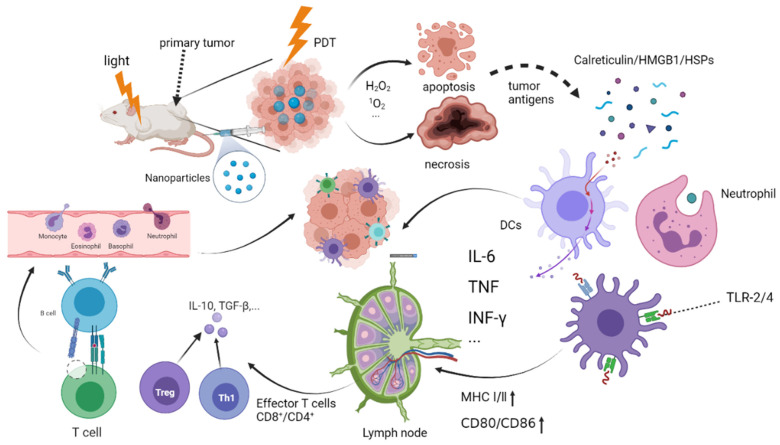
The mechanism of photodynamic triggering immunotherapy. Photodynamic therapy tumor cell apoptosis and necrosis, releasing tumor antigens and injury-related molecular patterns, including CRT, HSPs, and HMGB1. Activated CD4^+^ and CD8^+^ T cells are gathered in the lymph nodes and kill the tumor cells by inherent immunity and adaptive immunity. TLR-2/4: Toll-like receptor on the surface of the mononuclear macrophages; MHC Ⅰ/Ⅱ: Major histocompatibility class Ⅰ/Ⅱ.

A large number of studies have shown that tumor cells die through apoptosis and/or necrosis pathway [14,15] after PDT treatment. After undergoing apoptosis and/or necrosis, TAA was released, and the immune system was activated [16]. The acute inflammatory response after PDT treatment leads to the infiltration of host innate immune cells that eliminate any damaged cells [17]. This antitumor immune reaction is caused by PDT-inducing immunogenic cell death (ICD), which releases tumor antigens, injuring related gene expression, including calreticulin (CRT), after oxidative stress at the tumor site [18] (Figure 1). The production of ROS is essential as it activates intracellular signaling pathways that control the ICD [18]. Tumor cell evades the recognition and attack of the body’s immunity through adaptive immunity to realize self-protection. CRT is usually located in the endoplasmic reticulum (ER) and has an important effect as a chaperone molecule while maintaining the Ca2^+^ balance. CRT is embedded on the surface of the cell membrane to promote phagocytosis and secretion of interleukin-6(IL-6) as well as tumor necrosis factor (TNF); the latter is an essential proinflammatory factor [19]. Recent studies have shown that PDT induced by hypericin produces high ROS through ER preferential accumulation of photosensitive active substances, resulting in a stronger ICD [20]. In addition, high mobility group protein B1(HMGB1) released by dead tumor cells can bind to Toll-like receptors-4 (TLR4) receptors on the surface of DCs and promote antigen presentation while promoting the production of proinflammatory factors [21].

On the other hand, hot shock proteins(HSPs) [2] participate in the folding, assembly, intracellular transport, and degradation of related proteins in the form of molecular chaperones and promote cell viability by blocking cellular protein aggregation [22]. When cells are stressed by heat, heavy metals, ischemia, and toxins, HSPs, as the protective proteins, will be overexpressed to repair protein damage and prevent apoptosis. Among them, HSP70 and HSP90 have been shown to play a protective role against the apoptosis induced by PDT. As endogenous adjutants, some HSPs might stimulate adaptive immune responses [23]. HSP70 is a danger signal that can be identified by innate immune cells and then trigger the innate immune response [24]. At the same time, the DCs, the most effective APCs by far [25], are the bridge linking natural immune response and adaptive immune response. TAAs are transformed into immature T cells, which are then transformed into CTLs. This cascade of events removes residual tumor cells and initiates adaptive immunity. Shevsov et al. conducted experimental treatment on children aged 4.5-14 years who were diagnosed with brain tumors, and the results showed that the cluster of differentiation (CD)4^+^ and CD8^+^ T lymphocytes were increased and the regulatory T (Treg) cells were decreased in all 12 children. Serological analysis showed that Th1 cells and CD8^+^ T cells overexpressed IFN- γ and TNF-α while down-expressed the IL-10. Additionally, the data showed that HSP70 played a significant role in the adaptive immune process. Figure 1 shows the active process of PDT on tumor cells in vivo and its mechanism of stimulating immune cells and immune response [26].

### 2.2. The Basic Process of Photodynamic Immune Response

As a potential antigen pool, TAA is released by tumor cell fragments, and the immune system is activated. The whole process includes the following three steps antigen recognition stage, lymphocyte activation stage, and antigen clearance stage [9].

#### 2.2.1. Antigen Recognition Stage

This is an early stage of cellular immunity and encompasses the entrance of antigens into the body, recognition, presentation, and induction by the immune cells. The tumor cells that die from the PDT become TAAs. The released cell fragments and TAAs, such as CRT and HSP [27] are first phagocytized and then processed by local monocyte macrophages, B lymphocytes, DCs, and other immune cells [28].

#### 2.2.2. Lymphocyte Activation Stage

- Macrophages

**Figure 2:**
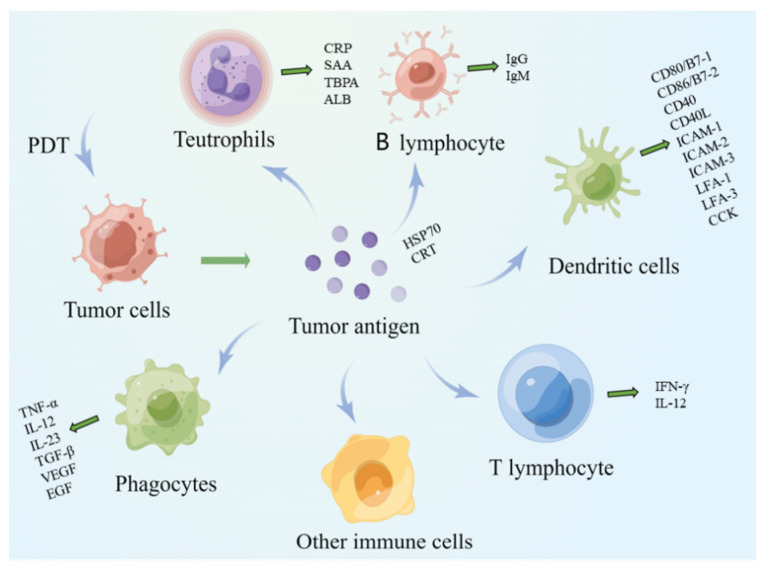
The process of immune response induced by tumor cells as antigens. Tumor cells produce tumor-associated antigens after PDT, which act on neutrophils, monocytes, macrophages, B cells, T cells, dendritic cells, and other immune cells. Immune cells are activated to produce cytokines to mediate the immune process in vivo.

Macrophages can absorb, process, and present TAAs. Major histocompatibility complex II (MHC II) molecules expressed on the surface of macrophages process antigen proteins into short peptides and then presented the information to helper T cells. Macrophages are classified into M1 type and M2 type according to the activation state and function. M1-type macrophages synthesize and secrete cytokines, including TNF-α, IL-12, and IL-23, promote the production of inflammation, and play host defense functions [29] (Figure 2). In contrast, tumor-related macrophages are M2 phenotype macrophages, which synthesize and secrete cytokines, including TGF-β, VEGF, and EGF, to reduce inflammatory response a mediate tissue repair processes, which promote tumor growth, invasion, and metastasis. After PDT, the proportion of macrophages with M2 phenotype characterization transforming into M1 phenotype increases, thus improving the adaptive immune response of the tumor cells [30,31].

- B lymphocytes

B lymphocytes secrete immunoglobulins obtain, process, and present tumor antigen information, enhance the tumor-killing effect of T cells, and induce a humoral immune response, which is the natural response of natural immune cells. After PDT, B lymphocytes produce IgG and IgM, which directly lead to tumor cell lysis [32].

- Dendritic cells

DCs are rich in antigen-presenting molecules such as MHC-I and MHC-II, costimulatory factors such as CD80/B7-1, CD86/B7-2, CD40, CD40L, as well as adhesion factors such as ICAM-1, ICAM-2, ICAM-3, LFA-1, and LFA-3. They are the most functional APCs in the body and can efficiently ingest and process tumor antigens [33]. Immature DCs have a strong ability to perform antigen phagocytosis. The DCs stimulated by tumor antigens, and other cytokines differentiate into mature cells, overexpress costimulatory factors and adhesion factors, and induce specific CTLs, thus achieving specific antitumor immune responses [34].

In addition, following PDT, the DCs highly express MHC-I and MHC-II molecules, bind with tumor antigens, form molecular complexes, present them to T cells, and start MHC-I restricted CTL reaction as well as MHC-II restricted CD4 ^+^ THL reaction. Additionally, the DCs secrete numerous costimulatory factors, including CD80/B7-1 and CD40, which are necessary cytokines for T-cell activation. DCs secrete chemokines, which increase the chemotactic aggregation of initial T cells, enhance the burst of T cells and the antitumor effect of effector T cells, and release antiangiogenic substances, including IFN-γ and IL-12, as well as inhibition of angiogenesis in tumor tissue. Because of the existence of feedback regulation, the DC-secreted chemokines enhance protein expression in DCs, which fuels antitumor immunity [35]. In addition, the DCs directly present tumor antigens to CTLs, which are activated with the help of activated CD4^+^ T cells. CTLs further enhance the body’s antitumor immune response by secreting cytokines or exerting direct killing. The DCs combine with T cells to induce cytotoxic T lymphocytes, cause Th1 immune response, activate perforin P, granzyme B, and FasL/Fas pathway and enhance NK cytotoxicity [36]. Thus, the DCs play a vital role in lymphocyte activation by participating in antigen information presentation and secretion of a variety of cytokines, such as costimulatory factors, adhesion factors, and chemokines.

- Natural killer cells

Natural killer (NK) cells are one of the most important immune cells in the body and are not limited by the MHCs or antibodies. The NK cells have non-specific recognition of target cells and widely participate in the body’s immune and antitumor regulation. Following PDT, there is a significant increase in the number of NK cells, which improves the antitumor efficacy of PDT conversely [37]. Exhaustion of NK cells in a mouse model was shown to cause tumor growth outside the scope of PDT [38]. Therefore, the NK cell plays an important role in improving the body’s immunity and inhibiting the growth activity of tumor cells.

- Neutrophils

Neutrophils are momentous for non-specific immune responses in blood. At the beginning of the immune response, neutrophils play a major role in the acute phase reaction [39]. At the same time, activating complement through alternative ways is the main factor causing early vascular injury. PDT treatment leads to acute phase reaction, an important period for the action of PDT in tumor treatment [40]. The three markers of the acute phase include the release of acute phase reactants, such as transferrin, albumin, elevated neutrophil levels, as well as activation of the pituitary/adrenaline axis [41].

Additionally, neutrophils are actively involved in the acute inflammatory phase reactions induced by the photodynamic response. Neutrophils can be induced into proliferation by mediators, such as chemokines, histamine, cytokines, and complement proteins [41]. These mediators have a significant impact on the efficacy of targeted tumor therapy [42]. In addition, while secreting cytokines, neutrophils are also regulated by cytokines secreted by other immune cells, which could regulate specific and non-specific immunity and confer certain antitumor effects.

#### 2.2.3. Antigen Clearance Phase

The antigen clearance stage is demonstrated when CTLs and antibodies inactivate and eliminate tumor antigens from the body. Under the action of cytokines, B lymphocytes secrete tumor antigen-specific antibodies and combine with tumor antigens to inactivate and eliminate the antigen. CTLs come into contact with tumor antigens and release a variety of cytokines, thus inducing immune inflammation that specifically binds and kills the tumor cells. At the same time, a combination of PDT and immune checkpoint inhibition could make immune cells with tumor antigen memory resist long-term tumor resistance [43]. Thus, the antigen clearance phase is pivotal in the immune process of tumor cells.

### 2.3. Innate and Adaptive Immune Responses in Anticancer PDT

The advantage of PIT is that it can provide not only effective solutions for the ablation of primary tumors but also local sources of tumor antigens and damage-associated molecular patterns (DAMPs), increase antigen presentation, enhance the immune effect, and achieve the goal of preventing tumor progression. The key that PDT can further induce immune response is that local activation of PSs can produce a large number of ROS-inducing chemical damage to tumor cells. Different intracellular locations of PSs will lead to different types of ICDs. As previously mentioned, apoptosis is caused by mitochondria, cell membrane destruction leads to necrosis, and autophagy is caused by lysosome/ER damage [44].

The location and principle of tumor tissue damage caused by different types of PDT are different. Local injury and oxidative stress of tumors induce acute inflammatory reactions, and a large number of white blood cells, including neutrophils, infiltrate local tumor tissues from blood vessels. Around tumor cells, tumor stromal cells, and damaged endothelial cells, PDT induces the rapid and large release of proinflammatory mediators and cytokines, further recruiting a large number of myeloid cells such as neutrophils. In addition, PDT activates antitumor immunity through the dangerous signal mechanism caused by the activation of DAMPs. The whole reaction process first stimulates and activates innate immunity, followed by the adaptive immune response [45].

Antitumor immune effects stimulated by PDT include the innate response of innate immune cells and the adaptive immune response regulated by T cells.

#### 2.3.1. Innate Response of Innate Immune Cells

After PDT-induced traumatic injury to the tumor cells, the first event that occurred at the PDT site was the production of DAMPs [46]. The DAMPs, key mediators of tumor cells’ immunogenicity killed by PDT via apoptosis or necrosis [17], are released after the tumor cell death induced by PDT and are recognized and detected by the innate immune cell [47]. The PDT-induced trauma to TME, as well as oxidative stress, stimulates the release of multiple proinflammatory regulators, such as TNF-α, complement proteins, IL-1, IL-6, arachidonic acid metabolites, and HSPs. In PDT-treated sites, innate immunocytes, including neutrophils and monocytes, were collected to attack damaged tumor cells [17]. Gollnick et al. used HPPH-PDT to treat BALB/c nude mice bearing Colo26 and demonstrated that low-energy excitation (48 J/cm^2^) significantly promoted local infiltration of neutrophils while maintaining the integrity of the blood vessels. Therefore, the inflammatory cells are necessary for the achievement of effective PDT effects. Neutrophils can also directly affect the proliferation and survival of CD8^+^ T cells [48]. Neutrophils can also directly affect the proliferation and survival of CD8^+^ T cells [7,49]. For instance, Kousis PC et al. showed that CXCR2^-^ with local neutrophil infiltration defect mice or neutrophil-depleted mice failed to survive after PDT treatment. The ability to produce a strong CD8^+^ cell-mediated antitumor immune response. Therefore, when PDT induces local inflammation, innate immunocytes play a central effect in affecting the proliferation and survival of CD4^+^ [50].

#### 2.3.2. Adaptive Immune Response

DCs are the bridge connecting innate immunity and adaptive immunity. As mentioned above, injured tumor cells express or release DAMPs, in which HSP70 bound to intracellular tumor-specific antigen is released to TLR2 and TLR4 on the surface of extracellular binding DCs to promote TLR maturation and movement to lymph nodes [35,51]. The mature TLR promotes the occurrence of adaptive immune response, and together with the innate immunity of the body, it acts on tumor cells to kill tumor cells [52]. HSP70, the main characterized PDT-induced DAMPs, is secreted after PDT [17]. Here, there is also upregulation of MHC I/II on the surface of the DCs. Additionally, CD4^+^ and CD8^+^ CTL are activated to cause early adaptive immune responses [17]. The activated CD4^+^ and CD8^+^ CTL play an antitumor role by inhibiting the division and proliferation of tumor cells in vivo [53].

Therefore, PDT is a very potent treatment in a cancer treatment option for cancer treatment. Through treatment activities, PDT can improve the immunogenicity of tumor cells, induce an adaptive immune response, and play a role in primary lesions and distant tumors.

## 3. The Methods to Improve PIT

The core components of PIT can mainly be divided into three parts, immune stimulators, tumors, and immune cells. Based on this, thus, improvement of immunochemotherapy can be realized through the introduction of immune stimulators in PDT-induced responses, epigenetic modification of TAA, as well as investigation of different immune cells for better anticancer effect.

### 3.1. Immune Stimulators

Immune activators are the substrates for evoking an organism to produce an immune response and are classified into two main categories, endogenous and exogenous, according to whether they derive from the organism itself or not.

Based on specific receptors on the surface of tumor cells, immune activators are regarded as “exosomes“, providing a powerful strategy to augment immune responses under the support of adjuvants and enabling the tuning of specific types of responses [54]. When the body’s immune system detects “exosomes“, an acute inflammatory response is rapidly induced, and a large number of leukocytes infiltrate locally in inflammation, after which more inflammatory factors and cytokines, including IL-6, TNF, ligands of TLR, etc., are released, allowing the activation of NK cells and DC cells, the latter through antigen presentation to activate B and T cells. This process is confined to the lymph nodes early on, as lymphocytes flow with the lymph, which in turn induces a systemic response.

#### 3.1.1. Exogenous Immune Stimulators

- Chitosan

Chitosan stimulates immune response effectively through different mediators, including TNF-α and IL-6. Animal models have demonstrated that it is feasible to boost immune responses through T-cell proliferation [55]. A new study conducted by Xiao Jun Cai et al. on multiple types of breast cancer tumor models concluded that the combined use of glycated chitosan (GC) and Photofrin in the process of PDT had good effects in inhibiting tumor growth, improving survival, and so on, owing to the ability of Photofrin PDT to enhance the local inflammatory response and attract immune cells to aggregate in the tumor area. The laser absorption dye used by the research group is Photofrin, and its aqueous solution has a primary absorption peak near 630 nm. The immune adjuvant is GC. First, the aqueous suspension of chitosan is incubated with three times of excess galactose, and then the mixture of Schiff base and Amadori product is stabilized by borohydride reduction to prepare the required compound [56].

Apart from chitosan, there are other polysaccharides as well that are known to stimulate immune response. Cyclodextrin and hyaluronic acid (HA) are two kinds of polysaccharides that have been widely studied. Cyclodextrins, as a class of cyclic oligosaccharides, have gained increasing attention for their applications in the preparation of macrocycles for various supramolecular prodrug systems because of their low price and strong biocompatibility [57]. Xiangyin Dai et al. designed a novel secondary assembly model of cyclodextrins, relying on the multivalent interactions between the main components, which can self-assemble in two stages to form uniform spherical nanoparticles (OF-NPs) with an average diameter of about 80 nm, and the photochromic properties can, in turn, be converted into ring closures (CF-NPs), accompanied by efficient energy transfer from donor 2 to CF-1 and gradual quenching of ^1^O_2_ generation [58]. HA, a negatively charged non-sulfated glycosaminoglycan is produced from D-glucuronic acid and by β Composition of bonded N-acetyl-D-glucosamine repeating units. HA and its derivatives are able to carry both water-soluble and water-insoluble active molecules in the form of liposomes. This characteristic can be used to form a coating layer for nanoparticles to enhance targeting ability. Soo Zeng Fiona Phua et al. reported a therapeutic system (HA-CAT@aCe6), including HA targeting CD44, catalase (CAT), and amantane-modified chloroprotein E6 (aCe6). Compared with the control system, significant tumor regression was observed in the experimental group after intravenous injection of the compound under light irradiation [59].

- CpG oligodeoxynucleotide

CpG oligodeoxynucleotides (CpG ODNs), which are synthetic unethylated DNA sequences mimicking bacterial DNA, have been confirmed to be able to recognize TLR9, further stimulate the organism’s immune response, whether it is innate or acquired immunity, thereby acting to inhibit tumor growth [60]. Yumin Xia et al. evaluated the effect of viteporfen-mediated PDT coupled with CpG ODNs on 4T1 metastatic breast cancer in a BALB/C immunocompetent mouse model after light irradiation at a wavelength of 690 nm for 15 min. The results showed a higher survival rate of mice following combination treatment compared to single treatment [61].

In addition, a large number of studies suggest that low-dose CpG ODNs have excellent immune adjuvant efficacy. For example, Atsushi Kajiwara et al. demonstrated that the combination of CpG ODNs and IL-4 enhanced Th1-specific immune response [62]. Ken J. Ishii et al. found that the combination of CpG ODNs and high-dose interleukin-13 Pseudomonas exotoxin (IL13-PE) had a significant inducing effect on long-term and complete tumor regression in nude mice [63]. More importantly, almost all studies mentioned that the antitumor effect of CpG ODNs was significantly higher than that of single drug application, which was conducive to the reduction of drug dose and the improvement of the curative effect.

- Nanoparticles

Nanoparticles (NPs) have been incorporated into PDT protocols due to their ability to deliver PSs into living organisms at reduced doses [64]. In the surface of a tumor integrin αvβ6-targeting peptide (the HK peptide)-functionalized graphene oxide (GO), Xinhe Yu et al. coated a layer of PS (HPPH) to construct a GO-(HPPH)-polyethylene glycol(PEG)-HK complex, which was shown to have the function in activating DCs by in vivo optical and single photon emission computed tomography (SPECT)/computed tomography(CT) imaging examinations in BALB/c mice, and the data showed that this complex could achieve the effect of preventing tumor growth and distant metastasis by promoting the infiltration of cytotoxic CD8^+^ T cells toward the tumor area [65]. The cross-immune reaction of the body’s own tissue components and the immune escape mechanism of tumor antigens are the biggest obstacles for the therapy against TAAs, based on which tumor-specific mutations become ideal antigens that are highly immunogenic and do not affect normal tissues [66]. PIT selectively focuses on tumors, and exposing specific antigens is another important direction for future PIT.

- Immune checkpoint inhibitors

Tumor cells often evade attacks by the body’s immune system in ways that directly or indirectly join immune checkpoints [67]. The role of immune checkpoint inhibitors (ICIS) has been recently demonstrated effective in a variety of cancers, and advances in cancer immunity represent a major breakthrough in the progression of cancer treatment [68]. Immune checkpoints, such as programmed cell death protein 1 (PD-1), cytotoxic T-lymphocyte-associated protein 4 (CTLA-4), and CD47, etc., are intrinsically inhibitors of the body’s immune system, and their mechanism of action is to produce an inhibitory signal after the binding of ligand proteins to immune checkpoints, which cannot be transmitted to immune cells to produce an effective immune response [57]. Bernhard kiss et al. designed and synthesized an ICI named after anti-cd47-ir700, and the team evaluated three human bladder cancer cell lines expressing CD47 mediated by this compound in the way of near-infrared photoimmunotherapy (NIR-PIT): UMUC3, 639V, and HT1376. Flow cytometry of propidium iodide was applied to assess the proportion of dead cells, with unirradiated cells and unlabeled anti-CD47 as controls. The results indicated that NIR-PIT increased cell death in a light dose-dependent manner in all three cell lines. At a maximum irradiation level of 40 J/cm^2^, more than 90% cell death was observed in 639V and UMUC3 cells, and more than 50% of HT1376 cells were dead. Meanwhile, the team also found that bladder cancer cells derived from fresh surgical specimens could detect up to 97.5% specific death by NIR-PIT at the highest energy level [69].

#### 3.1.2. Endogenous Immune Stimulators

- Low-dose cyclophosphamide

Cyclophosphamide is a common tumor-cytotoxic drug [70]. Long-term low-dose cyclophosphamide administration has been shown to effectively inhibit angiogenesis, which is essential for tumor growth and metastasis. Additionally, previous studies have shown that cyclophosphamide does not only significantly enhance the immunoreactivity of organisms, especially tumor-bearing CD8^+^ T-cells, to tumor antigens but also promotes the generation and release of CD34^+^ cells from the bone marrow. The CD34^+^ cells enhance the effect of granulocyte colony-stimulating factor (G-CSF) [71,72].

- Enzyme Stimuli

At present, proteases, phosphatases, kinases, lipases, and oxidoreductases are the most widely studied stimuli. Enzymes can process specific substrates, whereby probes and drug delivery systems can be designed that are capable of being acted upon by specific enzyme classes of the body. After the action, the signal of the body (such as fluorescence, photoacoustic, etc.) returns from the “off“ state to the “on“ state. On the one hand, the diagnosis of tumor tissue can be made; on the other hand, this effect contributes to the selective activation of therapeutic agents and the subsequent reduction of side effects [73].

Matrix metalloproteinases (MMPs) are zinc-dependent proteolytic metalloenzyme that mediates the degradation and remodeling of extracellular matrix highly expressed in metastatic tumor tissues. MMP-9 is one of the most complex and widely studied representative structures [74]. Based on this, the application value of MMPs as monitoring probes has been widely concerned. The research team of Douglas S. MacPherson designed three containing tyrosine or iodotyrosine, and their results showed that after using 3-iodotyrosine instead of tyrosine, the introduced halogen bond was able to induce structural changes within a single peptide, increasing the degree of intramolecular interactions. At the same time, the decrease in the packing degree of aromatic groups of peptides is able to reduce the intermolecular interactions. The two actions complement each other so that the reactive peptide is more hydrolyzed by MMP-9, and the released probe is ready for diagnosis [75].

- Granulocyte colony-stimulating factor and granulocyte-macrophage colony-stimulating factors

G-CSF, an endogenously synthesized hematopoietic cytokine in humans, can also be prepared by exogenous recombination. This immune factor, while increasing the chemotactic and phagocytic activity of neutrophils towards immunogenic sites, was able to enable reduced activation of apoptotic pathways [76]. Wil J. A. de Vree et al. investigated the role of IL-1 and G-CSF in PDT in rhabdomyosarcoma tumors. The study showed that the use of PDT enhanced the association between the level of IL-1 and the concentration of neutrophils at 4 h and the number of mature neutrophils at 8 h. It is further speculated that PDT can induce an increase in IL-1-dependent bone marrow neutrophil production rate. Additionally, in this process, IL-1 not only plays a role alone but also enhances the antitumor activity of neutrophils by stimulating the release of G-CSF [77].

Granulocyte-macrophage colony-stimulating factors (GM-CSF) is a hematopoietic growth factor produced by many cell types and, in turn, mediates the growth and differentiation of many cell types [78]. In addition, GM-CSF promotes angiogenesis by inducing the proliferation, migration, and differentiation of vascular endothelial cells [79]. It is because of the wide spectrum of effects that GM-CSF has been used in adjuvant tumor therapy [80]. For instance, Jinxie Zhang et al. employed colon-cancer-cell-derived neoantigen peptide adpgk co-encapsulated with black phosphorus quantum dots to form liposomes (Adpgk-BPQDs-liposomes) as a therapeutic vaccine and dispersed this liposome in a GM-CSF containing F127 gel. In this vaccine, black phosphorus generates so much heat that the ablation of F127 gel and release of GM-CSF are realized under the irradiation of an 808 nm near-infrared laser. The released GM-CSF then exerts the function that recruits APC cells and induces natural T cells. After the tumor-bearing mice were treated with the combination of PD-1 checkpoint blockade antibody and photothermal gel, the tumor development was significantly limited [81].

The success of endogenous tumor stimulators in blood cancers has gradually revealed the powerful role of immunotherapy in tumor treatment. With the US Food and Drug Administration (FDA) approval in 2017 having approved the first CAR-T cell therapy for the therapy of CD19-positive leukemia and lymphoma, research on endogenous tumor stimulators is constantly carried out, and immunotherapy is expected to be one of the breakthroughs in the diagnosis and treatment of tumors, but the problems of specific life-threatening toxicities caused by the complexity of immune mechanisms and the lack of tumor-specific targets have hampered the progress of research to some extent. The combination of targetability and biosafety of the therapy becomes an important approach to solving this problem. Another prominent advantage is that PIT is likely to achieve changes in the TME with strong targeting of immune escape mechanisms that are unique to the tumor [82,83].

### 3.2. TAAs

Since the discovery of tumor-specific antigens by Gross, there has been intense research on TAAs [84]. Among the molecular structures of TAAs are specialized peptide sequences capable of binding to MHCI/II molecules, and the binding triggers immune responses by antigen-specific CD8^+^ T lymphotoxin cells and CD4^+^ helper lymphocytes via recognition by CD4^+^ T cells [85]. Here, we present some common antigens that can be identified by PDT.

#### 3.2.1. HSPs

HSPs are a generally well-known class of molecular chaperones in genetics, for which naming is achieved by mass determination of a specific protein, such as HSP27, Hsp40, and Hsp60. Because they are associated with various cancer-related activities, such as cell proliferation, metastasis, and anticancer resistance, HSPs have become ideal candidates for PIT [86]. Mikako Ogawa et al. found that ICD, after being effectively induced by NIR-PIT, was able to destroy target cells bound to the antibody and photo absorber. Advanced living cell workstation revealed that NIR-PIT triggered irreversible damage to cell membrane function, resulting in cell swelling, rupture, and release of intracellular components. In addition, this process enables the relocation of corresponding ICD biomarkers, including CRT and HSPs, to the cell surface, and immunogenic signals such as Adenosine Triphosphate (ATP) and HMGB1 are rapidly transmitted to mature DCs [87]. Ahmad Jalili et al. studied murine colon carcinoma and showed that immature DCs significantly induced CTLs and NK cells through inoculating in tumor tissue therapied by PDT. Apoptosis and necrosis-related proteins such as Glucose-regulated protein 78(GRP78) and HSPs were significantly induced by PDT, which triggered cell apoptosis and necrosis.

Immature DCs cocultured with C-26 cells treated by PDT efficiently devoured tumor cells that were wounded by PDT, turned into mature DCs, and engendered a large amount of IL-12. The combined treatment of PDT and the administration of DCs produced an effective antitumor response [35]. PIT targeting HSPs is of better research value for tumor progression; on the one hand, this technology has the effect of inhibiting the action of HSPs acting as tumor growth-promoting factors; on the other hand, it is beneficial to consider the high expression of HSPs as biomarkers for the specific killing of tumor cells.

#### 3.2.2. Damage-Associated Molecular Patterns

In general, ICD is associated with the emission of a range of DAMPs generated in a precisely spatiotemporally defined configuration. In turn, DAMPs are characteristic of ICD. Under normal conditions, they are hidden inside cells and play roles in different physiological processes [88], but during cell death, DAMPs will be released by dying tumor cells or exposed to the cell surface. Yatim et al. classified DAMP as the type of constitutive DAMPs (cDAMPs), which was an internal molecule being expressed constitutively before death and was released by the dead cell and the type of inducible DAMPs (iDAMPs), relying on the potential way of cell death during the process of cell death [89,90]. This coordinated release allows the induction of a more robust antitumor-immune response with the establishment of immunological memory [91]. NIR light was able to kill tumor cells by binding single antibody conjugates of NIR activatable dyes (trastuzumab and panitumumab) to the membrane, whereas non-cell surface-bound conjugates had no effect [92]. Justyna MączyńSka et al. demonstrated that a novel human epidermal growth factor receptor-2(HER2) targeting conjugation conjugate, zher2:2395-ir700, causes HER2 specific cell death upon exposure to NIR light, leading to the release of DAMPs and activation of DCs in vitro [93].

#### 3.2.3. Cluster of Differentiation

CD44 is a surface cancer marker associated with drug resistance and is mainly responsible for intercellular adhesion, cell orientation, migration, and stromal cell signaling processes [94]. In addition, Foxp3^+^/CD25^+^/CD4^+^ Treg cells are vital to immunological self-tolerance and preventing autoimmune diseases [95]. NIR-PIT, coupled with CD44^−^ and CD25 targeted agents, has the potential to directly eliminate tumor cells directly by removing Foxp3^+^CD25^+^CD4^+^ Treg cells from the TME to amplify the immune response. Yasuhiro maruoka et al. demonstrated that a NIR-PIT combination has the role in significant tumor growth inhibition and prolonged survival compared to CD44 targeted NIR-PIT alone, and in MC38 Luc and LL/2 tumors, showed prolonged survival compared to CD25 targeted NIR-PIT alone [96].

### 3.3. Immune Cells

#### 3.3.1. Tumor Associated Macrophage

Tumor associated macrophages (TAMs), one of the major tumor-infiltrating immune cell types, are divided into two different polarization states: M1-type macrophages and M2-type macrophages [97]. In terms of interactions with tumors, the two forms of macrophages confer opposite effects. The M1 macrophages have antitumor functions, such as direct cytotoxicity and antibody-dependent cell-mediated cytotoxicity (ADCC) of tumor cells [98]. In contrast, the M2 macrophages accelerate the tumorigenesis and metastasis of tumor cells, restrain the T-cell-regulated antitumor immune response, as well as promote tumor angiogenesis, thus leading to tumor progression [99]. Xiaosong Zhang et al. designed a hyaluronic acid-modified multifunctional black phosphorus (HA-BP) nanoparticle immune cell regulator targeted polyethylenediphosphine. The diameter of HA-BP nanoparticles is 56 nm. The zeta potential of BP modified with mPEG-NH_2_ changed from −23.7 mV to −6.03 mV and then decreased to −28.4 mV after HA functionalization and through the results on murine monocytic macrophage leukemia cell line RAW264.7 cells, HA-BP decreased the level of CD206 by 42.3%, while increased the expression level of CD86 by 59.6%, suggesting that HA-BP nanoparticles achieved the effect from protumoral M2 TAMs to antitumor M1 macrophages by changing the cellular phenotype [100].

#### 3.3.2. Neutrophils

Generally, neutrophils are supposed to be one of the earliest cells in the innate immune response to enter the tumor site treated by PDT [101]. Upon entry, they secrete various factors, such as lysosomal enzymes, neutrophil-derived myeloperoxidase (MPO), and reactive oxygen species (ROS), which have the propensity to destroy residual tumor cells and the vasculature [102]. Qiujun Qiu et al. elaborated a novel strategy for cancer immunotherapy that promotes tumor infiltration of neutrophils by PDT or PIT via delivery of ibrutinib (IBR) nanocomposites. With hydrogenated soybean phosphatidylcholine and CH as the lipid components of PDT/PTT, DiR liposomes were prepared by an improved ethanol injection method. IBR was loaded and transported to the mouse model at the center of the liposomes. By studying the effect of this complex on breast cancer-bearing mice, Qiujun Qiu et al. found that IBR-mediated immunization therapy combined with DiR-mediated PDT/PIT had several advantages, whose synergistic interaction resulted in marked efficacy in the treatment of breast cancer [103].

#### 3.3.3. T Lymphocytes

T cells are key mediators of tumor destruction. Notably, they specifically target antigens expressed by tumors and possess various intrinsic properties, such as persistence, longevity, and function, which enable them to be effective immunotherapeutic agents [104]. A new PIT technology utilizing ferritin-loaded PSs, the duty of an anti-fibroblast activation protein scFv to act as a targeting ligand, was developed by Zipeng Zhen et al. Ferritin is regarded as a compact nanoparticle protein cage. Using this property, the research group used ferritin as a PS carrier and coupled FAP specific single chain variable fragment (scFv) to the surface of ferritin to obtain nano conjugates, Z@FRT-scFv. This FAP-targeted PIT was able to efficiently and selectively eliminate cancer-associated fibroblasts (CAFs) from the tumor, and this process had no systemic toxic effects. Following the scavenger of CAFs, this technique was continued to destroy the extracellular matrix and reduced secretion of CXCL12, the latter of which can significantly enhance CD8^+^ T cell infiltration, ultimately eliciting a corresponding immune response leading to effective cancer cell death [105].

Recent advances in immunotherapies targeting immunosuppressive cells, such as Tregs, have been highlighted in tumor treatment. It has long been recognized that Treg can inhibit the production of effective protective antitumor immunity, thereby inhibiting the function of effector T cells, which will contribute to cancer progression to a certain extent [106]. Therefore, the elimination of immunosuppressive Treg function through PDT may be crucial to trigger an effective antitumor immune response. Eleonora Reginato’s research group studied the immunological changes induced by PDT in patients with invasive esophageal squamous cell carcinoma and the effects of PDT on the level and function of Tregs. The level of CD4^+^CD25^+^CD127^−^ FoxP3^+^ Treg in blood samples of patients before and after PDT was detected by flow cytometry. The functional Tregs were detected by co-culture and proliferation test with T effector cells. They found that although PDT had no effect on Treg level, it eliminated the inhibition of peripheral Tregs [107].

## 4. Application of Photodynamic Immune Response

We previously detailed several PDT-based immune responses, including the mechanisms of PIT action as well as its drawbacks and improvements. Based on this information, it is evident that PIT has great potential for cancer treatment, both during the early and late stages of development. Different mechanisms through which PIT achieves cancer treatment are highlighted in subsequent sections.

### 4.1. Photoimmunotherapy

Although PIT is similar to PDT in that it relies on PSs to exert its antitumor activity, it does not use ordinary PSs. Notably, PSs bind to monoclonal antibodies or their fragments to target antigenic clusters of tumor cells, thereby enhancing tumor-targeting efficiency [108]. Numerous studies have applied different types of PSs to bind various monoclonal antibodies [109]. The results demonstrated that PIT exerted a killing effect on tumor cells, suggesting that it may be an effective anticancer agent [108].

Most PSs applied in PDT are amphiphilic substances, which are apt to gather in aqueous solutions. However, antibodies are water-soluble molecules; thus, PS immune conjugates contain free PS impurities. To this end, it is difficult to distinguish the targeted effect of PS immune conjugates from the non-specific effects of free PS impurities [110].

With regards to PS’s ability to bind to monoclonal antibodies and form PS immune complexes, Savellano and Hasan identified an effective conjugate of BPD Verteporfin and an antibody C225 (epidermal growth factor receptor extracellular domain chimeric antibody) using an antibody PEGylation and a 50% dimethyl sulfone-50% water dual solvent system. The former significantly increased the solubility of PS to reduce the aggregation of PS, and the latter is to prevent the non-covalent action of PS and PS aggregation. As far as the molecular structure of the compound is concerned, the preparation of its purity is the key to realizing its role. The results of the spectral analysis showed that with the increase in photosensitizer immunoconjugates (PICs) loading rate, the protein absorption peak at 280 nm of the prepared PIC with BPD–Ab molar ratio of 2 gradually decreased in an expected way compared with the PS absorption peak. However, for PIC with BPD–Ab molar load ratio greater than 10, the yield decreases to less than 45%. In addition, when attempting to increase the molar loading rate of BPD–Ab to about 11, the residual free PS impurities of the PIC preparation increased to more than 10% even after extensive efforts to purify the conjugate. It was found that although the obtained PS immune conjugate could kill EGFR overexpressed cells by PDT, the effect was not as good as that of BPD alone [111].

Previous studies have shown that PS binds to monoclonal antibody fragments to form a PS immune complex. For example, Bhatti et al. used PPA to bind a variety of scFvs, which were coupled with activated PSs in dimethyl sulfoxide and acetonitrile solution [112]. Notably, scFvs retained their solubility in the solution, while the hydrophobic PSs could stay in the solution for a long enough time to improve reaction efficiency. The authors also found that C6.5-PPA had an IC50 (drug concentration required for 50% in vitro inhibition) that was 70-fold higher than that of free PPA, while that of mFE-PPA was 7 times higher than that of free PPA [112]. The position and quantity of lysine determine the suitability of scFv as a PS carrier. Through the experimental exploration and testing of the research group, the number of lysines that can be used for coupling is 6, two of which are adjacent to each other in the sequence, and the ratio of PS:scFv is 2.1:1, which indicates that the two PS on scFv are more appropriate in the form of coupling. The results also showed that scFV-PPA binding was cleared faster compared with PPA in vitro. This indicates that the time of exposure to light can be shortened, making it more treatment-appealing, with little risk of skin photosensitivity. This will guide the future design of better PS immune complexes.

PIT may be an effective way of cancer treatment, which provides a new idea for cancer treatment. In the experiment, the solubility of PS was increased in various ways to reduce the deviation caused by free PS impurities. Although both antibodies or their fragments can form immune complexes with PS for PDT, the latter produces a superior effect. At present, an antibody-conjured drug (ADC) composed of Cetuximab and IRDye700DX, has been approved by the Ministry of Health, Labor, and Welfare in Japan. In addition to ASP-1929, Rakuten is currently developing several other drugs, including ASP-1929 in combination with PD-1, which has entered clinical Phase I trials.

### 4.2. PDT Combined with Adaptive Immunotherapy

The combination therapy with PDT and specific immunity may be a preferred method for cancer treatment, and this therapy is easier to administer compared to PIT [108]. DCs, which harbor antigen-presenting properties, are the most effective inducers of adaptive immunity. Jalili et al. applied immature DCs in combination with photofrin-PDT in mice with colon cancer [35] and found that PDT creates a unique environment for the release of tumor antigens and “danger” signals that may cause DCs maturation.

In addition, tumor cell lysates produced by PDT have been shown to activate DCs and induce an antitumor immune response [113]. Inoculation of immature DCs into PDT-treated tumors enabled the effective migration of these cells to local and distal lymph nodes and stimulated the cytotoxic activity of lymphocytes isolated from local lymph nodes [35]. Numerous studies have shown that PDT combined with immunomodulators can enhance the antitumor effect [114,115].

Combination therapy results in an enhanced antitumor response that is not limited to the tumor being treated but is also effective in controlling distant growth. PDT postoperative tumor injection of DCs may have several advantages, including load reduction in in vitro tumor antigens, elimination of injection of DCs by other means that cannot predict the concerns of the trafficking and allowing the DCs in the case of continuous inflammation acquisition, processing and rendering tumor source material. This may make the whole process more immunogenic [35]. However, the feasibility of this treatment strategy required future explorations in a clinical setting.

Antiangiogenic drugs can inhibit the growth of tumor blood vessels, thus cutting off the nutrient supply of the tumor, inhibiting the growth of tumor cells, and playing the anticancer role, which can assist PDT in providing an additional anticancer activity. For example, Jiang et al. used photofrin-PDT in combination with monoclonal antibodies (MF1 and DC101) to treat vascular endothelial growth factor (VEGF) receptors in intracranial glioblastoma in mice [116]. In this study, the researchers did not choose to manufacture a composite preparation of photosensitive materials and monoclonal antibodies. Instead, they chose the scheme of simultaneous photodynamic therapy and intravenous injection of vascular endothelial growth factor inhibitors in tumor-bearing mice. The experimental results showed that the combined therapy could inhibit the angiogenesis of glioblastoma, resulting in a reduction in tumor size and prolonging survival rates, and the anticancer efficacy of the combined therapy was superior to those of PDT alone or anti-angiogenesis drugs. Similar results were also obtained using monoclonal antibodies against VEGF in combination with hypericin-PDT in the treatment of mouse bladder cancer tumors [117].

In addition, photofrin-PDT decreased the expression of decay-accelerating factor (DAF, CD55), Protectin, and complement-receptor-1-related protein y(Crry) on SCCVII cells in mice, thereby making the cells susceptible to complement deposition and effective clearance by phagocytes [118]. The researchers injected the mice with antibodies against Crry, Protectin, or DAF, after PDT and found that PDT combined with anti-Crry and anti-protectin resulted in higher tumor suppression rates compared with PDT alone. Conversely, PDT combined with anti-DAF resulted in the opposite effect, possibly due to the additional role played by DAF in T-cell signal transduction.

In summary, PDT, in combination with adaptive immunotherapy, generates a superior effect than PDT alone. Notably, the former achieves the purpose of treating cancer by stimulating the cytotoxic activity of lymphocytes and enhancing the antitumor immune response. Moreover, PDT can also be used in combination with anti-vascular drugs, and this has been shown to generate a superior effect than PDT or anti-vascular drugs alone.

### 4.3. Cancer Vaccines

Traditional vaccines introduce diluted or killed microorganisms that the body recognizes as foreign antigens and produce protective antibodies [119]. PDT reportedly induces and enhances antitumor immune responses associated with effective recognition of tumor antigens [120], a phenomenon that has generated speculation about its potential as an anticancer vaccine [121].

Cancer vaccines work by exposing tumor cells to a lethal dose of radiation and then introducing these killed tumor cells or tumor cell lysates into animals where the host’s immune system will recognize the foreign “antigen” and produce immunity [122]. Unlike PDT, in cancer vaccine applications, PS is neither administered to the host nor is the tumor exposed to light [108]. In direct contact with the host, the vaccine was composed of autologous dead tumor cells or cell lysates treated with PDT [123].

Sandra O Gollnick et al. comparing the cancer vaccine potential of PDT-generated tumor cell lysates with those generated by ultraviolet or ionizing radiation [124], found that PDT-generated vaccines exhibited tumor specificity and induced CTLs responses compared with other methods [119].

The superior efficacy of PDT-generated lysates may be attributed to the fact that they stimulate phenotypic and functional maturation of DCs better than lysates obtained by other means [113]. Moreover, Korbelik and Sun produced a whole-cancer therapeutic vaccine [121] by expanding mouse SCCVII cells with BPD in vitro, followed by exposure to light. Injection of this vaccine around established subcutaneous SCCVII tumors yielded significant therapeutic effects, including growth retardation, regression, and cure, and vaccinated mice recorded a significantly higher number of cells across all major lymph node groups relative to unvaccinated counterparts [108].

Furthermore, Korbelik’s and Sun’s relevant studies also demonstrated that the proximity of the vaccination site to the treated lesion was associated with treatment outcomes. For example, although injecting PDT at the distal end of the tumor was still effective, the effect was less than that of peri-focal treatment [125].

**Figure 3:**
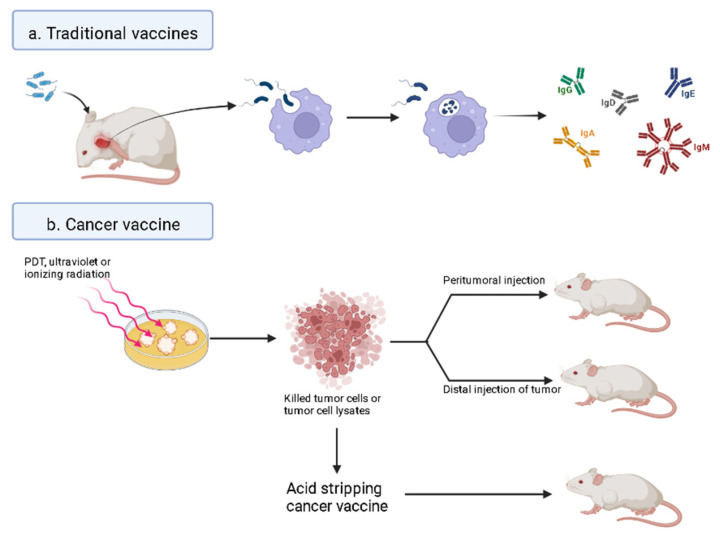
Cancer vaccines. (**a**) Traditional vaccines. The body recognizes foreign invading pathogens, activates the immune system, and produces a large number of antibodies to kill the pathogens. (**b**) Cancer vaccines. PDT induces killed tumor or cell lysates, production of vaccines using antigenic peptides for delivery back into the body through the injection around the tumor or the remote injection of the tumor to activate the immune system.

Apart from this whole-cell PDT vaccine, Shixiang et al. investigated a PDT-based acid-stripping cancer vaccine (Figure 3), which is produced by exposing C6 cells to HMME-PDT and acid stripping by exposing DCs to C6 glioma cancer cell antigen polypeptides [126]. Demonstrated in their report a more desirable preparation method for tumor cell vaccines, namely using acid elution after PDT treatment, which has the characteristic of generating tumor-specific cell surface antigens before adoptive transfer. Antigenic peptides interacting with MHC-I factors on the tumor cell membrane were acid eluted, whereas antigens interacting with MHC-II (ox-6) or other non-MHC factors were unaffected. This method does not affect the structure of the polypeptide. Therefore, the antigenic peptide prepared by the PDT acid elution method can immunize DCS more effectively, leading to the production of DC vaccines. Flow cytometry analysis showed that the expression of mature cell markers CD80 and ox-6 was increased in antigen-treated dendritic cells obtained from the PDT acid elution and freeze-thaw groups compared with control dendritic cells, to some extent, reflecting the enhanced function of DCS. The results showed that PDT-generated antigens, which were further purified by pickling, had the greatest stimulative effect on DCs, evidenced by elevated serum IL-12 and TNF-α as well as decreased serum IL-10 levels. This PDT-based pickling vaccine appeared to be more effective than the whole-cell PDT vaccine [108].

Previous studies have shown that vaccines made of inactivated tumor cells or tumor cell lysates obtained by PDT exhibit tumor specificity, can induce CTLs responses, and effectively kill cancer cells. However, vaccines made from cancer cell lysates are more efficacious. Additional research evidence has also shown that the proximity of the injection site to the treatment focus affects treatment outcomes. Overall, vaccines based on PDT acid elution have superior efficacy to whole-cell ones, although further explorations are needed to elucidate the precise effects.

## 5. Conclusions

Compared with the traditional tumor treatment methods of chemotherapy, radiotherapy, and surgery, PDT, because of its non-invasive, low systemic toxicity, and tumor-targeting specificity makes the treatment more efficient and shows broader prospects in clinical application. PDT can not only destroy the primary tumor but also trigger immune reactions to prevent its subsequent metastasis and diffusion, thereby conferring immunomodulatory properties [104]. Although PDT-induced immune response is hard to define, owing to the complexity of the TME and involvement of numerous cytokines and immune cells, it is clear that PDT furnishes effective immune induction and generates better results by maximizing local inflammatory responses as well as activating immune cells to destroy tumor tissue.

The formation of the PS immune complex via PS binding to monoclonal antibodies enables enhanced tumor targeting of PSs, a combination of antiangiogenic drugs can enhance the anticancer effects, and PDT-generated vaccines are tumor-specific and induce CTLs responses. Currently, some drugs have gradually completed many clinical trials and shown excellent anticancer efficacy, strongly suggesting the clinical utility and feasibility of these emerging treatment modalities and drugs in cancer management. Photodynamic immunotherapy, obtained by combining PDT with immunotherapy, has shown promise in improving antitumor immune response, which not only inhibits tumor spread and metastasis but also exerts long-term immune memory, realizing synergistic treatment as well as improving therapeutic efficacy and cure rate. Approaches, such as the application of immuno-stimulators that enhance PDT-induced responses, TAA improved by epigenetic modification, and investigation of different immune cells, are expected to guide the combination of phototherapy and immunotherapy for better anticancer effects. Furthermore, with the gradual deepening of PIT research and its continuous maturity in tumor combination therapy, tumor-targeted PIT has great potential to become widely used first-line tumor therapies.

Despite the better antitumor effect of combination treatment of PDT with immunotherapy, PIT still has some limitations. First, there are insufficient experimental and clinical studies of PIT so investigators cannot provide reliable evidence for clinical application. Second, not all details are understood; more studies are needed to comprehensively elucidate the mechanisms by which PIT improves therapeutic efficacy. In addition, the strength and controllability of the immune response must also be addressed, which needs further investigation. This new pattern is expected to be a crucial supplement to the development of traditional tumor-treatment strategies. Although obstacles remain, there are valuable and encouraging opportunities in the PIT field for the foreseeable future.

## Data Availability

Data sharing not applicable.

## Abbreviations

Abbreviations	The full name
PS	PS
PDT	Photodynamic Therapy
PIT	Photoimmunotherapy
ROS	Reactive Oxygen Species
mAb	Monoclonal Antibody
DC	Dendritic Cell
CRT	Calreticulin
HSP	Heat Shock Proteins
HSP70	Heat Shock Proteins 70
HSP90	Heat Shock Proteins 90
HMGB1	High Mobility Group Protein 1
ER	Endoplasmic Reticulum
MHC	Major Histocompatibility Complex
MHCⅠ/Ⅱ	Major Histocompatibility Class Ⅰ/Ⅱ
TNF	Tumor Necrosis Factor
TNF-α	Tumor Necrosis Factor-A
TA-As	Tumor-Associated Antigens
DCs-α	Dendritic Cells A
IL	Interleukin
IL-6	Interleukin- 6
IL-12	Interleukin-12
IL-23	Interleukin-23
TLR4	Toll-Like Receptors-4
Treg	Regulatory T
TGF-β	Transforming Growth Factor Beta
VEGF	Vascular Endothelial Growth Factor
EGF	Epidermal Growth Factor
ICAM-1	Intercellular Cell Adhesion Molecule-1
ICAM-2	Intercellular Cell Adhesion Molecule-2
ICAM-3,	Intercellular Cell Adhesion Molecule-3
LFA-1	Lymphocyte Function-Associated Antigen-1
LFA-3	Lymphocyte Function-Associated Antigen-3
APCs	Antigen-Presenting Cells
CTLIFN-γ	Cytotoxic T LymphocytesInterferon-Γ
DAMPs	Damage-Associated Molecular Patterns
cDAMPs	Cell-Death-Associated Molecular Patterns
TRL2/4	Toll-Like Receptors 2 And 4
CRP	C Reactive Protein
SAA	Serum Amyloid A
TBPA	Transferrin-Binding Protein A
ALB	Albumin
CCK	Chemotactic Cytokines
CD80/B7-1	Recombinant Human Activation B7-1 Antigen
CD86/B7-2	B-Lymphocyte Antigen B7-2
CD40	Clusters Of Differentiation 40
CD40L	Clusters Of Differentiation 40 Ligand
CD91	Low-Density Lipoprotein Receptor-Related Protein 1
ODN	Oligodeoxynucleotide
DNA	Deoxyribonucleic Acid
TLR9	Toll-Like Receptor 9
NPs	Nanoparticles
GO	Functionalized Graphene Oxide
HK	His-Lys
HPPH	Photochlor
SPECT	Single Photon Emission Computed Tomography
CT	Computed Tomography
CD	Cluster Of Differentiation
PD-1	Programmed Cell Death Protein 1
CTLA-4	Cytotoxic T-Lymphocyte-Associated Protein 4
G-CSF	Granulocyte-Colony Stimulating Factors
GM-CSF	Granulocyte Macrophage-Colony Stimulating Factors
FDA	The Us Food and Drug Administration
HSP	Heat Shock Protein
HMGB-1	High-Mobility Group Protein 1
ATP	Adenosine Triphosphate
HER2	Human Epidermal Growth Factor Receptor-2
TAMs	Tumor-Associated Macrophages
ADCC	Antibody-Dependent Cell-Mediated Cytotoxicity
HA	Hyaluronic Acid
BP	Black Phosphorus
MPO	Myeloperoxidase
IBR	Ibrutinib
DiR	Dialkylcarbocyanine 1,1’-Dioctadecyl-3,3,3’,3’-Tetramethylindotricarbocyanine Iodide
CAFs	Cancer-Associated Fibroblasts
CXCL	Chemokine (C-X-C Motif) Ligand
MMP	Matrix Metalloproteinases
PPa	Pyropheophorbide-a
scFvs	Single-Chain Fv Fragments
ADC	Antibody Conjured Drug
VEGF	Vascular Endothelial Growth Factor
DAF	Decay-Accelerating Factor
HMME-PDT	Hematoporphyrin-PDT
ICD	Immunogenic Cell Death
